# EANM position paper on the role of radiobiology in nuclear medicine

**DOI:** 10.1007/s00259-021-05345-9

**Published:** 2021-04-29

**Authors:** An Aerts, Uta Eberlein, Sören Holm, Roland Hustinx, Mark Konijnenberg, Lidia Strigari, Fijs W.B. van Leeuwen, Gerhard Glatting, Michael Lassmann

**Affiliations:** 1 Radiobiology Unit, Institute for Environment, Health and Safety, Belgian Nuclear Research Centre (SCK CEN), Mol, Belgium; 2 Department of Nuclear Medicine, University Hospital Würzburg, Würzburg, Germany; 3 Department of Clinical Physiology, Nuclear Medicine and PET, Rigshospitalet, University Hospital Copenhagen, Copenhagen, Denmark; 4 Division of Nuclear Medicine and Oncological Imaging, University Hospital of Liège, GIGA-CRC in vivo Imaging, University of Liège, Liège, Belgium; 5 Department of Radiology and Nuclear Medicine, Erasmus MC, Rotterdam, The Netherlands; 6 Medical Physics Department, IRCCS Azienda Ospedaliero-Universitaria di Bologna, Bologna, Italy; 7 Interventional Molecular Imaging Laboratory, Department of Radiology, Leiden University Medical Center, Leiden, The Netherlands; 8 Medical Radiation Physics, Department of Nuclear Medicine, Ulm University, Ulm, Germany

**Keywords:** Radiobiology, Dosimetry, Biodosimetry, Biomarkers, Radionuclide therapy

## Abstract

With an increasing variety of radiopharmaceuticals for diagnostic or therapeutic nuclear medicine as valuable diagnostic or treatment option, radiobiology plays an important role in supporting optimizations. This comprises particularly safety and efficacy of radionuclide therapies, specifically tailored to each patient. As absorbed dose rates and absorbed dose distributions in space and time are very different between external irradiation and systemic radionuclide exposure, distinct radiation-induced biological responses are expected in nuclear medicine, which need to be explored. This calls for a dedicated nuclear medicine radiobiology. Radiobiology findings and absorbed dose measurements will enable an improved estimation and prediction of efficacy and adverse effects. Moreover, a better understanding on the fundamental biological mechanisms underlying tumor and normal tissue responses will help to identify predictive and prognostic biomarkers as well as biomarkers for treatment follow-up. In addition, radiobiology can form the basis for the development of radiosensitizing strategies and radioprotectant agents. Thus, EANM believes that, beyond in vitro and preclinical evaluations, radiobiology will bring important added value to clinical studies and to clinical teams. Therefore, EANM strongly supports active collaboration between radiochemists, radiopharmacists, radiobiologists, medical physicists, and physicians to foster research toward precision nuclear medicine.

## Introduction

In recent years, the number of radiopharmaceuticals for diagnostic and therapeutic applications has increased considerably. In addition, theranostics are being developed that use the same molecular targeting platform for both imaging and treatment. This has led to an increased medical use in both malignant as well as benign conditions. Consequently, an improved understanding of the biological processes, with special regard to the effects of ionizing radiation to normal tissues and tumors, is required. This is to determine the absorbed dose-effect relationship more precisely, as a prerequisite for achieving an optimal diagnostic or therapeutic outcome. In general, absorbed doses are low (< 20 mGy) for most organs in diagnostic procedures [1]. However, when multiple diagnostic examinations or therapeutic applications are undertaken, this is no longer the case. Repeated diagnostic irradiations can result in cumulative absorbed doses in normal organs and tissues up to a few hundred milligray [2]. For therapies, absorbed doses can exceed previously suggested absorbed dose limits (e.g., 23 Gy for the kidneys in peptide receptor radionuclide therapies [3]). For absorbed doses of < 1 Gy, stochastic effects of ionizing radiation may be observed, whereas for therapies, a mixture of stochastic and deterministic effects is expected. Radiation-related adverse effects strongly depend on both the individual absorbed doses [4] and the individual radiation sensitivity [5–8]. Thus, without an individualized approach in radionuclide therapy, a group of patients may be over-treated, jeopardizing patient safety. Conversely, patients may be undertreated, leading to suboptimal treatment efficacy.

Systemic radiation delivery via radiopharmaceuticals is inherently different from irradiation by external radiation sources. As a consequence, distinct radiation-induced biological responses are expected for radiopharmaceuticals posing considerable challenges for in vitro, preclinical and clinical studies investigating radionuclide applications. Several research topics are suggested that should be addressed with regard to radiobiology in relation to the systemic use of radiopharmaceuticals and which have not yet been rigorously investigated [9–11]. Presently, there are limited studies related to the use of radiobiology in nuclear medicine. Typical examples of such studies are provided in Table 1.

**Table 1:** Non-exhaustive list of typical examples of nuclear medicine radiobiology studies including a description of the method or biomarker applied, targets, radionuclides, and activity/absorbed dose range

Topics investigated	Method/biomarker	Target	Radionuclide	Model	Activity/dose range	Remark	References
DNA damage	ɣ-H2AX, 53BP1 foci in PMBCs	Blood	^177^Lu, ^131^I, ^223^Ra	Patients	< 100 mGy	Ex vivo and in vivo data	[7, 12–16]
DNA damage	ɣ-H2AX, 53BP1 foci in tumor cells	Neuroendocrine tumor, (SST_2_), prostate tumor (PSMA)	^177^Lu, ^213^Bi	Cell culture, mouse	< 2.5 MBq ^177^Lu in vitro, 30 MBq ^177^Lu in vivo, 0.3 MBq ^213^Bi in vitro, < 6.6 MBq ^213^Bi in vivo	SST_2_ agonist vs. antagonist	[17, 18]
DNA damage Imaging	[^111^In]In-anti-γH2AX-TAT, [^89^Zr]Zr-DFO-anti-γH2AX-TAT	Neuroendocrine tumor, pancreatic carcinoma	^177^Lu, ^225^Ac	Mouse	< 20 MBq ^177^Lu, 37 kBq ^225^Ac	DNA damage monitoring after [^177^Lu]Lu-DOTA-TATE therapy	[19, 20]
Preclinical therapeutic value, cell survival, cell cycle progression	Tumor volume, cell survival, cell cycle analysis	Non-Hodgkin lymphoma (CD37)	^177^Lu	Cell culture, mouse, patient samples	< 6 MBq/mL ^177^Lu in vitro, < 500 MBq/kg ^177^Lu in vivo	Radioimmunotherapy	[21]
In vitro cytotoxicity	Cell-free plasmid DNA damage, DNA damage, cell survival, cell viability, microautoradiography cell distribution assay	Breast cancer (HER2), prostate tumor	^67^Ga, ^111^In	Cell culture	< 0.3 MBq/mL ^67^Ga in vitro; 1.1 Bq/cell (15 MBq/mL) ^67^Ga or ^111^In in vitro, 0.1 MBq/mL ^67^Ga or ^111^In cell free	Auger electrons	[22, 23]
Combination with other agents: radiosensitizing agents	53BP1, micronuclei in cell cultures, cell survival, cell viability, cell cycle progression, DNA damage response, gene expression, tumor perfusion, tumor, tumor radioactivity uptake, tumor volume	Neuroendocrine tumor (SST_2_), small cell lung cancer (SST_2_), prostate tumor (PSMA), neuroblastoma PARP, protein folding, DNA and DNA synthesis, Hedgehog signaling, nicotinamide phosphoribosyltransferase, topoisomerase I, proteasome, P53-MDM2 interaction nutlin-3, and the copper-chelated form of the oxidizing agent disulfiram, G2/M cell cycle arrest	^177^Lu, ^131^I	Cell culture, spheroids, mouse, patients	< 6 MBq/mL ^177^Lu in vitro, < 30 MBq ^177^Lu in vivo, < 6 MBq ^177^Lu ex vivo patients, 4 × 7.8 GBq patients, 0.37 MBq/mL ^131^I in vitro, 20 MBq ^131^I in vivo	Olaparib, 1,5-dihydroxyisoquinoline, PJ-34, veliparib, talazoparib, Hsp90 inhibitor, androgen receptor inhibitor, capecitabine, temozolomide, sonidegib, NAMPT inhibitor, topotecan, bortezomib, the inhibitor of the P53-MDM2 interaction nutlin-3 and the copper-chelated form of the oxidizing agent disulfiram (DSF:Cu), EBRT	[21, 24–37]
Combination with other agents: upregulation of the therapeutic target	Transcriptional, translational, and functional analysis, tracer uptake	Neuroendocrine tumor (SST_2_)	n.a.	Cell culture	n.a.	–	[38, 39]
Combination with other agents: chemotherapeutic drugs	Cell viability, biodistribution, tumor volume	Breast cancer	^131^I	Cell culture, mouse	< 7.4 MBq/mL in vitro, 7.4 MBq in vivo	Human serum albumin–paclitaxel nanoparticles	[40]
Combination with other agents: radioprotectant agents	Biodistribution, tumor response	Kidneys	^177^Lu	Mouse	30 MBq	Kidney-preserving agent	[41]
Tumor radionuclide/receptor distribution	[^111^In]In-EGF and [^111^In]In-labeled trastuzumab imaging, autoradiography, immunofluorescence microscopy	Breast cancer (EGFR, HER2), head and neck cancer (EGFR), neuroendocrine tumor (SST_2_)	^111^In, ^177^Lu	Spheroids, cell culture, mouse, patients (ex vivo)	1 MBq/mL ^177^Lu in vitro, 30 MBq ^177^Lu in vivo	–	[42, 43]
Molecular profiling	Blood NET transcript analysis	Neuroendocrine tumors (SST_2_)	^177^Lu	Patients	[^177^Lu]Lu-DOTA-TATE-based PRRT	NETest, PPQ: PRRT predictive quotient (PPQ)	[44–46]
Molecular profiling	Whole genome microarray analysis	Neuroendocrine tumor (SST_2_), thyroid gland, various normal tissues, Kidney	^177^Lu, ^131^I, ^211^At	Mouse, rat	< 15 MBq ^177^Lu, < 4.7 MBq ^131^I, < 42 kBq ^211^At	–	[47–50]
Molecular profiling	Targeted next-generation sequencing of DNA damage-repair associated genes	Prostate cancer	^225^Ac	Biopsies	[^225^Ac]Ac-PSMA-617 therapy	–	[8]
Relative biological effectiveness	Cell survival	Neuroendocrine tumor (SST_2_)	^177^Lu, ^213^Bi	Cell culture	< 10 Gy (^177^Lu), < 5.2 MBq (7 Gy) ^213^Bi	RBE = 6	[51]
Radiation quality effects	Cell survival, cell viability, gene expression, DNA damage, in vivo therapy studies		^131^I, ^161^Tb, ^177^Lu	Cell culture, mouse	< 9.25 MBq ^131^I in vitro	–	[52, 53]
Cell membrane-mediated non-targeted effects	Cell membrane lipid raft analysis, underlying signaling pathways, cell survival, DNA damage tumor volume	Colon cancer (CEA), vulvar squamous carcinoma (A431 HER2 + CEA), ovarian carcinoma (SKOV3 MISRII), endometrial carcinoma (AN3CA MISRII)	^125^I, ^212^Pb/^212^Bi, ^213^Bi	Cell culture, mouse	< 0.5 MBq/ml ^212^Pb in vitro, 0.5 MBq/mL ^213^Bi in vitro, < 4 MBq ^125^I in vitro, 1.48 MBq ^212^Pb in vivo, 37 MBq ^125^I in vivo	–	[54, 55]
Single cell and micrometastases dosimetry	Calculation	Neuroendocrine tumor (SST_2_)	^177^Lu, ^161^Tb	Cell culture, computed cell model	2.5 MBq/mL ^177^Lu in vitro	–	[56, 57]
Radiobiology, generic dose models	Calculation	Kidneys, tumor				Development of the linear-quadratic model for nuclear medicine	[58–64]
Thyroid dose-toxicity model	Retrospective calculations of TCP, NTCP	Thyroid treatment	^131^I	Patients	< 560 MBq [65]	Retrospective analysis	[65, 66]
Hepatocellular carcinoma tumor response	Prospective study based on [^99m^Tc]Tc macro-aggregated albumin dosimetry	Liver treatment glass microspheres	^90^Y	Patients	> 205 Gy	Prospective study	[67]
Hepatic dose-toxicity model	BED, TD, EUD	Liver treatment glass and resin microspheres	^90^Y	–	< 250 Gy BED_50_	Dose-toxicity model	[68]
Kidney dose-toxicity model	BED, TCP, NTCP	SST_2_ agonists, treatment of neuroendocrine tumors	^90^Y, ^177^Lu	Patients	40 Gy BED	Clinical trial	[69]
Kidneys and red bone marrow toxicity model	BED	Neuroendocrine tumor	^177^Lu	Virtual patients	40 Gy_2.5_ kidneys BED, 2 Gy_15_ red bone marrow BED	In silico clinical trial	[70]
mIBG treatment	Retrospective calculations	Neuroblastoma mIBG treatment	^131^I	Patients	30 GBq	Two fractions	[71, 72]
Predicting tumor response	BED	Prostate carcinoma	^177^Lu	Patients	7.3 ± 0.3 GBq	Prediction of tumor volume shrinking using PBPK/PD modeling	[73]

*BED* biologically effective dose, EBRT external beam radiation therapy, *EUD* equivalent uniform dose, *NAMPT* nicotinamide phosphoribosyltransferase, *NTCP* normal-tissue complication probability, *PMBC* peripheral mononuclear blood cells, RBE relative biological effectiveness, *SST*_*2*_ somatostatin receptor subtype 2, *TCP* tumor control probability, *TD* tolerable dose, *PARP* poly-[ADP-ribose]-polymerase 1, *PRRT* peptide receptor radionuclide therapy

In addition, in a recent review it was stated that, for radiation oncology, incomplete physics and dosimetry reporting limits the progress of radiobiology [74]. The authors concluded that there is not only a crucial deficiency on experimental details but also a lack of interaction between medical physicists and radiobiologists. The reporting of results pertaining to radiobiology in nuclear medicine is often provided in activities rather than absorbed doses (Table 1). Therefore, future radiobiology studies in nuclear medicine will benefit from including good practice of dosimetry reporting [75]. Conversely, dosimetry calculations should be based on available experimental biological data.

Consequently, in nuclear medicine, patient care optimization principles, which include not only absorbed dose or dose rate parameters but also radiobiological parameters, should be integrated. To this end, close interaction and collaboration between radiochemists, radiopharmacists, radiobiologists, medical physicists, and physicians will be needed.

## Radiobiology

Radiobiology (also known as radiation biology) is a branch of biology concerned with the biological effects of ionizing radiation on living organisms. Radiobiology studies the interactions of ionizing radiation on atomic and molecular structures and consequently their induced effects on cells, tissues, and organs, both normal and diseased. As such, radiobiology enhances the understanding of biological outcome (harm or benefit) from ionizing radiation exposure.

**Fig. 1:**
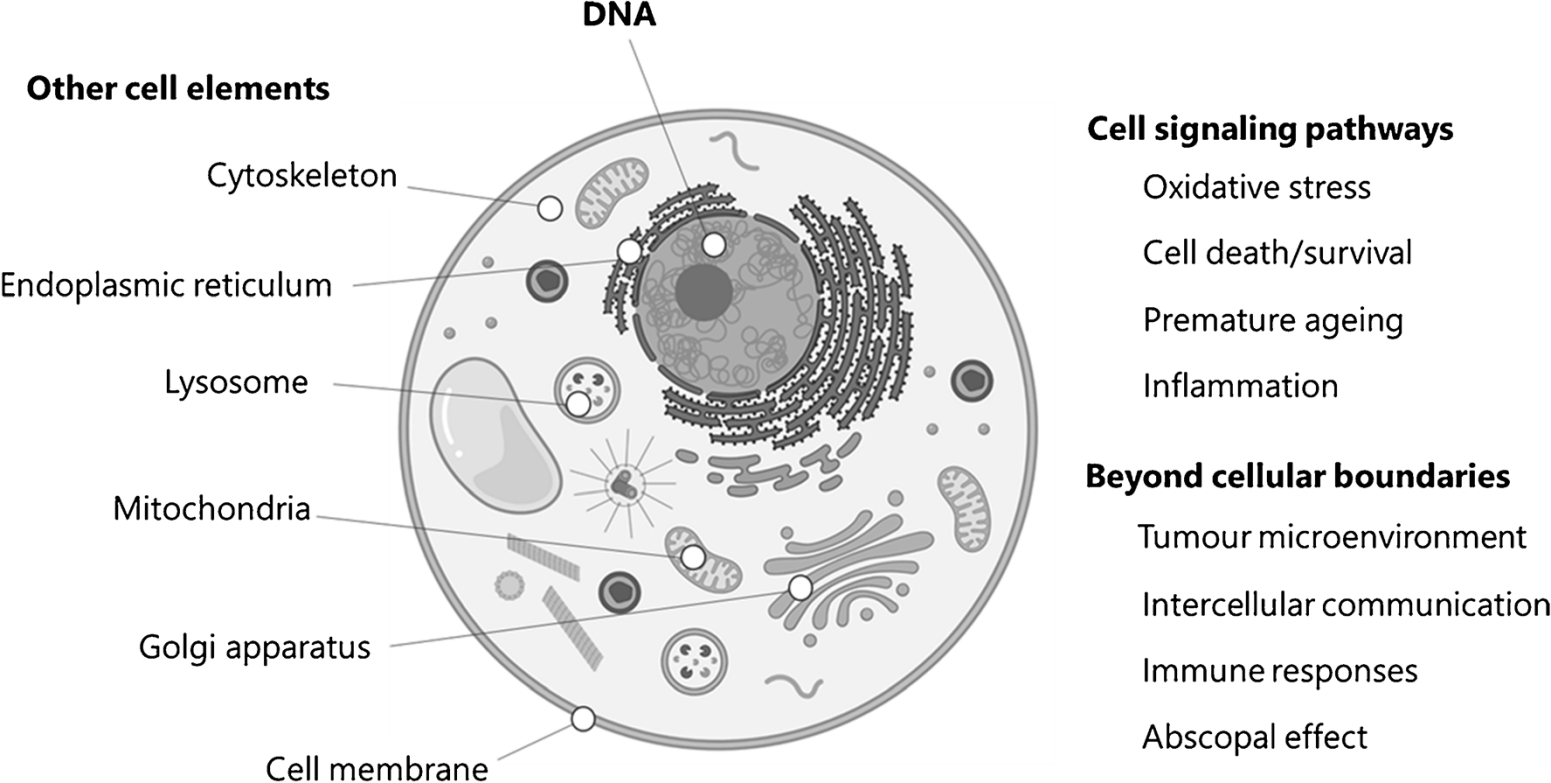
Interaction of ionizing radiation with cellular matter, DNA, and much more. DNA and other cell elements as potential targets for ionizing radiation damage. Ionizing radiation also impacts cell signaling pathways like oxidative stress, cell death and survival pathways, premature aging, and inflammation, all of which moreover are highly interconnected. Also, aspects beyond the cellular boundaries must be considered, like intercellular communication, the tumor microenvironment, the immune system, and the abscopal effect. Image created using BioRender.com

When ionizing radiation impinges living matter, it deposits energy along its path leading to atomic ionization, thereby damaging biological molecular structures (Fig. 1). In the common paradigm, DNA is considered the critical target for radiation damage [76]. However, not only DNA, but also proteins, lipids and metabolites may be modified by ionizing radiation [77, 78]. In direct action, absorption of ionizing radiation will happen at the site of the atoms of the cellular molecules. Subsequent ionization events may cause breakage of chemical bonds. It may also convert atoms and molecules into free radicals with very reactive unpaired electrons that can further react with neighboring molecules after which a chain of damaging reactions may occur. The indirect effect from the absorption of ionizing radiation is the production of free hydroxyl and other highly reactive radicals, due to the hydrolysis of water molecules. Despite their short existence, they can still diffuse to and damage other cellular molecules. Moreover, oxygen can create reactive oxygen or nitrogen radical species with greater stability, longer lifetimes, and thus wider diffusion possibilities [76, 78]. The abundance of these oxygen radicals generates a condition known as oxidative stress which can further impact cellular signaling and alter metabolic pathways resulting in, among others, cell death mechanisms, senescence, and inflammation [78, 79]. Furthermore, ionizing radiation can harm supramolecular structures like cellular membranes, mitochondria, the endoplasmic reticulum, the Golgi apparatus, the lysosomal system, and the cytoskeleton [80]. Finally, also aspects beyond cellular boundaries are increasingly being considered in radiobiology, like the tumor microenvironment, intercellular communication, immune responses, and the abscopal effect [81, 82].

Research in radiobiology can rely on the newest techniques and insights in biology in general and is exploiting (epi)genomics, proteomics, metabolomics, high-throughput screening, and exploring new models like stem cells, organoids, in vivo orthotopic and subcutaneous patient-derived xenograft models, or siRNA- or CRISPR/Cas9-derived models. These are anticipated to lead to new hypotheses to understand the effects generated from ionizing radiation on biological systems and to improve therapies based on ionizing radiation [78, 83].

Today, improved insights into the dose-response effects caused by ionizing radiation on tumor cell killing as well as on acute and long-term normal tissue collateral damage are impacting greatly treatment planning in external beam radiation therapy (EBRT) [83, 84]. Of several models, the linear-quadratic (LQ) model has been best validated by experimental and clinical data to describe cell survival fractions. The selection of accurate LQ parameters, α, β, and α/β, is pivotal for a reliable estimate of radiation response. Clinically, the LQ model is mainly used to estimate equivalent radiotherapy schedules (e.g., calculate the equivalent dose in 2-Gy fractions), but increasingly also to predict tumor control probability (TCP) and normal tissue complication probability (NTCP) using logistic models [76, 85]. In addition, radiobiological discoveries are guiding clinical trials that test EBRT combined with inhibitors of the DNA damage response and immune or cell cycle checkpoint inhibitors. To have maximum impact for individual patients, predictive biomarkers should be identified that enable the rational selection of treatments to combine with EBRT. Further research into the radiobiology of tumor metabolism, cancer stem cells, and the tumor microenvironment has the potential to translate current knowledge and future gains to the clinic [83].

The question arises whether there is a need for a dedicated nuclear medicine radiobiology or whether we can rely on radiobiological models derived for EBRT or brachytherapy.

The response of a living system to an irradiation strongly depends on the distribution of absorbed doses across space and time. As these dose distributions in EBRT and brachytherapy are very different to those in radionuclide therapy, extrapolation of EBRT or brachytherapy radiobiology to radionuclide therapy is not straightforward. Indeed, the specific physical characteristics of radionuclide therapy (mixed radiation qualities, time-varying and protracted exposure, low absorbed dose rates, and inhomogeneous dose distributions) differ from those of conventional EBRT (short exposure time, high absorbed dose rate, and mostly homogeneous irradiation fields) and brachytherapy (even in the low dose rate case there is a well-defined source distribution). As a result, the responses of irradiated tissues and of the human/patient are expected to be different for radionuclide therapy [10, 86, 87]. For example, due to the time-varying and comparatively low dose rates in radionuclide therapies, the DNA damage induction and repair may strongly differ compared to EBRT [7, 12, 13, 75]. Consequently, there is a need for the generation and application of more radiobiological knowledge specific for nuclear medicine diagnostic and therapeutic procedures.

Efforts to gather more evidence in radiobiology regarding systemic exposure to ionizing radiation in nuclear medicine applications have been increasing recently; this is illustrated in this paragraph at the example of [^177^Lu]Lu-DOTA-[Tyr3]octreotate ([^177^Lu]Lu-DOTA-TATE) radionuclide therapy in patients with advanced, progressive, somatostatin receptor subtype (SST_2_)-positive midgut neuroendocrine tumors (NETs) that was studied in the NETTER-1 phase III trial [88]. Even though [^177^Lu]Lu-DOTA-TATE is clearly successful in terms of survival benefits, current figures could be further improved. In addition, treatment is limited by potential adverse effects on the kidneys and the bone marrow, hindering the use to its full potential. This emphasizes the need to further optimize [^177^Lu]Lu-DOTA-TATE radionuclide therapy to further improve efficacy while reducing toxicity. This includes improved dosimetry hand in hand with a deep biological evaluation of superior radionuclides, improved SST_2_ ligands, increased SST_2_ levels, the role of tumor microenvironment, and combinations with immunotherapy, targeted therapy or DNA modulating agents, as well as predictive markers for improved patient selection and treatment follow-up [24, 44, 45, 89–91].

## Position of the EANM

Radiobiology is particularly relevant for nuclear medicine therapies, as these treatments differ substantially from irradiation by external radiation sources. This is highlighted in the common strategic research agenda for radiation protection in medicine [92] developed by the five medical societies involved in the medical application of ionizing radiation, which later founded the European Alliance for Medical Radiation Protection Research (EURAMED) (https://www.euramed.eu/)^1^. An improved understanding of the biological processes with special regard to the effects of ionizing radiation to normal tissues and tumors is needed to individualize the use of existing and future developed radiopharmaceuticals. Therefore, the radiobiological knowledge concerning the specific needs of nuclear medicine (e.g., patient-specific and tumor-specific radiation sensitivity, dose-effect relationships, spatio-temporal properties, therapy response, normal tissue effects, role of microenvironment and systemic reactions, combination therapies) must be obtained and considered together with physical and medical parameters in the development of nuclear medicine procedures. This will also foster the principles stated in the EC Directive 2013/59/Euratom, article 56, that exposures of target volumes in nuclear medicine treatments shall be individually planned and their delivery appropriately verified [93]. How to interpret the EC directive for nuclear medicine therapies is further detailed in the recently published EANM position paper on article 56 of the Council Directive 2013/59/Euratom [94].

Absorbed dose measurements can be combined with radiobiological parameters to enable an improved estimation and prediction of efficacy and adverse effects, which can further support treatment planning [94]. This additional input is presently, however, very rarely used, as only limited studies related to therapeutic use of radiopharmaceuticals and including radiobiological parameters are available. Of note, the precision dosimetry approach to describe the dose on the cellular and subcellular level in targeted radionuclide therapy is under development [56, 57].

In diagnostic nuclear medicine applications, especially in longitudinal procedures, the role of radiobiology and the long-term consequences of radiobiology-related findings, such as reported in studies on DNA damage and repair, still has to be defined [95–101]. Currently, these studies provide no evidence that diagnostic nuclear medicine procedures are not safe.

Consequently, EANM believes that, to further optimize nuclear medicine procedures for each individual patient, efforts should be undertaken to promote the integration of radiobiology within nuclear medicine by endorsing further research and teaching activities. The knowledge of different disciplines, such as biology, chemistry, medicine, pharmacy, and physics, can then be combined for providing reproducible results, which are, ideally, traceable to metrological standards.

### Essential radiobiological studies

**Fig. 2:**
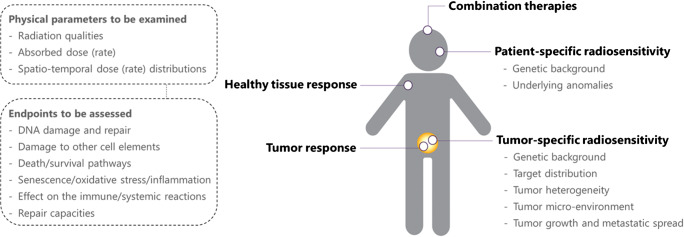
Contributions of radiobiology to nuclear medicine. Radiobiology helps to understand patient- and tumor-specific radiosensitivities. In addition, radiobiology is fundamental to a mechanistic understanding of the therapeutic capacity of nuclear medicine agents and their potential short- and long-term toxicities, including the dose–effect relationships herein. Biological data will serve as input for dosimetry, together leading to a more accurate estimation of efficacy and adverse effects. Ideally, this will lead to patient-specific dosing schemes. Moreover, further fundamental knowledge about the biological mechanisms underlying tumor and healthy tissue responses will help in identifying predictive and prognostic biomarkers as well as biomarkers for treatment follow-up. In addition, it can form the basis for the development of combination therapies, including radiosensitizing and radioprotectant strategies. Image created using BioRender.com

The nature of radiation exposure resulting from nuclear medicine procedures is diverse and comprises different radiation qualities, absorbed doses, dose rates, and temporal and spatial dose distributions [102, 103]. Low doses are encountered in diagnostic procedures as well as from out-of-target therapeutic exposures. High doses are expected within the tumor and in the close proximity of the tumor during radionuclide therapy. The determination of the absorbed dose to the tissue and on a (sub-)cellular scale are a prerequisite for defining dose-effect relationships, both in estimating (pre-)clinical therapy outcome and normal tissue toxicity as well as in assessing the cellular and molecular mechanisms, including repair capacity (Fig. 2).

With the ambition to maximize the benefits of radiopharmaceutical products that are effective and safe for each individual patient, preclinical and translational science undertakes dedicated research to understand the biological characteristics of tumor and normal tissue intrinsic radiosensitivities and the fundamental biological mechanisms underlying the therapeutic and short- and long-term cytotoxic effects of radiopharmaceutical products, as well as determining the dose-effect relationship herein (Fig. 2) [9–11]. Essential aspects to investigate related to patient- and tumor-specific radiosensitivities include genetic background and underlying anomalies that impact radiosensitivity, target distribution, tumor heterogeneity, tumor micro-environment, and tumor growth and metastatic spread. Important endpoints to be assessed in tumor and healthy tissue responses are DNA damage, damage to other cell elements, death and survival pathways, oxidative stress and inflammation, effect on the immune and systemic reactions, and repair capacities. To this end, high-end molecular and cellular biology tools, omics data analyses (proteomic, transcriptomic, genomic, radiomics) as well as bioimaging (microscopy, PET/CT or SPECT/CT, autoradiography) are available. As such, radiobiological data may reveal patient-specific radiation sensitivity traits useful as predictive biomarkers of response for a personalized radionuclide therapy regimen (e.g., genomic traits, target level and distribution, anomalies in signaling pathways altering radiosensitivity) as well as biomarkers useful for therapy response monitoring, both on a therapeutic aspect as well as for normal tissue damage. Radiobiological findings may also be used as input for the development of radiosensitizers or radioprotectant agents. Moreover, the radiobiology of fractionation schemes (how many/how much activity per fraction) as well as the radiobiology of combination therapies (combinations of radionuclide therapy with chemotherapy, tyrosine kinase inhibitors, immunotherapy, hormone therapy, or radiosensitizers) [9, 25] is not well explored and could be helpful in defining optimal treatment strategies. Finally, standardization of biological study protocols as well as depositing study data in repositories is required to allow comparison and combining of cohorts.

Some publications, regarding both external and internal irradiation, indicate that there is a very low dose range (< 10 mGy) which shows a different dose response compared to higher doses [99, 104–107]. Therefore, extrapolating from higher absorbed doses and dose rates to very low doses and dose rates is not straightforward and needs further research. It is likely that other, different, biological responses exist after low-dose and high-dose ionizing radiation exposure using radiopharmaceuticals for both tumor and normal tissues.

### Investigating low-dose radiation effects in nuclear medicine

The linear-no-threshold (LNT) model based on the extrapolation of epidemiological data at high absorbed doses is currently used to estimate the risk at low doses [108], although this is a matter of debate [109, 110]. An important aspect of the justification of using this model is that radiation carcinogenesis has been assumed to be primarily driven by the damage to the DNA and subsequent mutation of growth-regulating genes in target cells. Yet, a number of other potential mechanisms contributing to and modulating radiation carcinogenesis have been proposed, including epigenetic mechanisms of gene regulation such as DNA methylation and miRNA expression, transmissible genomic instability, bystander effects, and adaptive response. The extent to which these modulating effects and non-mutational mechanisms challenge the validity of the LNT risk extrapolation model needs to be determined. For this purpose, the use of well-validated animal and human cellular/tissue models of radiation carcinogenesis (both solid cancers and leukemias) is required. In addition, also non-cancer effects (e.g., cardiovascular and neurocognitive) should be considered and studied [111].

The key question here is whether the LNT model is valid for internal radiation exposure such as that encountered from nuclear medicine procedures with typically low dose rates, heterogeneous dose distributions, and a protracted nature of exposure. Therefore, the determination of corresponding low-dose-effect relationships can be a basis for risk assessment also in radiation protection (ALARA, LNT hypothesis, second cancer risk), e.g., in medical imaging or staff exposure.

To describe and monitor such effects, studies are needed to identify biomarkers for assessing short-term or medium-/long-term stochastic radiation risks (cancer and non-cancer) that (1) are sufficiently sensitive in the low-dose range (< 100 mGy), (2) are strongly linked to medium-/long-term side effects of ionizing radiation, and (3) possess definite dose/dose rate/dose fractionation/radiation quality dependencies.

### Investigating therapeutic radiation effects in nuclear medicine

Currently, dose-effect relations are not fully utilized in most radionuclide therapies, as these therapies are given at a minimal activity that is deemed safe in all patients and effective to some extent. This often results in suboptimal therapy delivery. Radioembolization therapies arguably form the exception to this rule, as both normal liver thresholds and tumor target absorbed doses are considered as input to the treatment planning of these therapies.

More studies should be undertaken to determine TCP and NTCP curves for specific radionuclide therapies. In this context, radiobiological data (e.g., LQ α/β parameters and repair kinetics) can serve as input for better dose-effect modeling [58, 59, 65, 70] taking into account the radiation quality, dose rate, dose fractionation, and dose distribution on the tissue as well as on the (sub)cellular scale [60]. Finally, results from comparison studies with external beam radiation therapy could inform on better treatment strategies in nuclear medicine.

Radiobiology and dosimetry should be integrated in all stages of the development of individualized radionuclide therapy drugs. Preclinical experiments should deliver the radiobiological data through standardized and controlled settings with multiple cancer models to study response variability. Radiobiological concepts should ideally form the basis for the design of clinical trial protocols. Phase I studies focus on safety and thus should consider both absorbed dose and individual patient radiosensitivity. Phase II studies should ideally be based on absorbed dose thresholds and individual radiosensitivity. In many cases, the choice is made for a phase II trial with fixed activity at the maximum tolerable activity from phase I in order to simplify the clinical protocol. However, without absorbed doses available, it is impossible to build knowledge on dose-effect relationships and prospective clinical trials based on individually absorbed doses are crucial [67].

## Discussion

Radiobiological knowledge is not yet used in many nuclear medicine applications, or it is used only in a basic phenomenological manner, such as that integrated in the model of biologically effective dose (BED). This can be attributed to two main reasons:

1. Detailed radiobiological knowledge is currently not readily available, because of

- The specifics of nuclear medicine procedures and their multiple parameters involved (dose, dose rate, individual DNA repair capacity, …) as well as the heterogeneity of the conditions being treated.
- The complexity of its integration into the clinical procedures for example due to technical constraints (e.g., microscopes, bone marrow biopsies) or missing know-how.
- The associated patient load (time per patient per measurement).
- The resources and costs associated (personnel, supplies, and others).

2. The phenomenological parameters (LQ α/β parameters, DNA repair) known from EBRT are incorrectly thought to suffice fully for the needs of all nuclear medicine applications.

All of the above points need to be addressed for continued improvement of nuclear medicine procedures, including the adequate integration of radiobiology. To solve item 1.a requires intensified research as previously discussed, while item 1.b needs education and training of all involved, scientists and physicians. Both call for the need of standardized procedures to produce reliable data. Lastly, items 1.c and 1.d are an issue for cost-benefit analysis which is mandatory for all medical procedures. This applies also to item 2 whose applicability and justification should be applied adhering to the requirements of best available science and cost-benefit analysis.

Thus, to develop an optimal nuclear medicine procedure one needs to acquire and include all knowledge and appropriate commitment from all involved in the process. This will inevitably include time investment from the patient for more measurements. Such collection of data and inclusion of a priori knowledge is the required input for a rigorous cost-benefit analysis. Implemented therapies will then be well founded, both scientifically and in terms of cost and effort.

## Conclusion

While the role of radiobiology for diagnostics remains to be clarified, there is a clear role for radiobiology in optimizing the benefits of therapeutic radiopharmaceuticals to ensure that they are effective and safe for each individual patient. Just as radiobiology data are routinely used in EBRT treatment planning, nuclear medicine could also benefit from a deeper integration of such data. Therefore, there is a need to better define the dose-effect relationships of systemic ionizing radiation for tumors as well as for normal tissue. As absorbed dose rates and absorbed dose distributions in space and time are very different between external irradiation and systemic radionuclide exposure, distinct radiation-induced biological responses are expected in nuclear medicine and need to be explored. It is expected that a better understanding of radiobiological parameters can contribute to fully exploit the capabilities of new and existing nuclear medicine applications to be effective and safe for each individual patient. To this end, a strong link between radiochemists, radiopharmacists, radiobiologists, medical physicists, and physicians is warranted to design sound study designs. In particular, the inclusion of radiobiologists in the clinical team will be advantageous.

## Footnotes

1 EURAMED represents the European Association of Nuclear Medicine (EANM), the European Federation of Organizations for Medical Physics (EFOMP), the European Federation of Radiographer Societies (EFRS), the European Society of Radiology (ESR), and the European Society for Radiotherapy and Oncology (ESTRO).
